# Evaluation of the Effect of Copper Sulfate Exposure on Organs in Juvenile Rats

**DOI:** 10.3390/ijms27125542

**Published:** 2026-06-19

**Authors:** Osman Öztürk, Seher Yılmaz, Aslı Okan, Sümeyye Uçar, Emin Kaymak, Evrim Suna Arıkan Söylemez, Şükrü Ateş, Taha Berkay Bor, Züleyha Doğanyiğit

**Affiliations:** 1 Department of Internal Medicine Sciences, Faculty of Medicine, Yozgat Bozok University, Yozgat 66100, Turkey; 2 Department of Anatomy, Faculty of Medicine, Yozgat Bozok University, Yozgat 66100, Turkey; 3 Department of Histology and Embryology, Faculty of Medicine, Yozgat Bozok University, Yozgat 66100, Turkey; 4 Department of Anatomy, Faculty of Medicine, Erciyes University, Kayseri 38030, Turkey; 5 Department of Medical Biology, Faculty of Medicine, Afyonkarahisar Health Sciences University, Afyonkarahisar 03000, Turkey

**Keywords:** copper sulphate, pesticide, rat, oxidative stress, inflammation

## Abstract

Copper sulphate pentahydrate is widely used in agriculture to control bacterial and fungal diseases in various crops. Despite its extensive application, limited data exist regarding its potential toxic effects on juvenile rats following early-life exposure. In addition to oxidative stress and inflammation, copper overload may also trigger cuproptosis, a recently identified copper-dependent form of regulated cell death. This study aimed to investigate the histopathological, biochemical, and molecular effects of copper sulphate exposure on major organs in juvenile rats and to elucidate the associated inflammatory and oxidative stress-related mechanisms. Male and female Sprague–Dawley rats (30–40 days old, 50–70 g) were randomly assigned to control and experimental groups. Following copper sulphate exposure, histopathological examinations were performed on major organs, including the liver, kidney, heart, lung, and reproductive tissues (testis in males and ovary in females). Immunohistochemical analyses of tumor necrosis factor-alpha (TNF-α) and nuclear factor kappa B (NF-κB) were conducted. Oxidative stress parameters, including malondialdehyde (MDA), total antioxidant status (TAS), and total oxidant status (TOS), were measured using ELISA. Gene expression levels of TNF-α and NF-κB were evaluated by quantitative real-time PCR (qRT-PCR). Copper sulphate exposure induced significant histopathological alterations in all examined tissues of both male and female juvenile rats. Biochemical findings revealed increased oxidative stress, evidenced by elevated MDA and TOS levels along with altered TAS values. Furthermore, immunohistochemical and gene expression analyses demonstrated upregulation of TNF-α and NF-κB, indicating activation of inflammatory pathways. Copper sulphate exposure leads to widespread morphological changes in juvenile rats, potentially mediated by oxidative stress and inflammation. These findings provide insight into the biological impact of early-life pesticide exposure. Further studies are warranted to clarify the underlying molecular mechanisms and to develop effective preventive or therapeutic approaches.

## 1. Introduction

Copper (Cu) is an essential trace element involved in numerous physiological and biochemical processes, including hematopoiesis, mitochondrial energy production, antioxidant defense, immune regulation, and the synthesis of neurotransmitters and neuropeptides [1,2,3]. Owing to these biological functions, copper has been widely used in pharmaceuticals, industrial products, and, more recently, in agricultural applications such as antimicrobial coatings, pesticides, and feed additives [1,2,3]. Among copper-containing compounds, copper sulfate pentahydrate is extensively used worldwide, including in Türkiye, as a fungicide and bactericide to control bacterial and fungal diseases in crops, fruits, and vegetables. However, excessive exposure to copper sulfate may lead to copper accumulation in tissues and subsequent heavy metal toxicity [4,5].

Under physiological conditions, cellular copper homeostasis is tightly regulated by specialized transport systems, including the high-affinity copper transporter 1 (CTR1), which mediates copper uptake, and the P-type ATPases ATP7A and ATP7B, which control intracellular trafficking and efflux to prevent toxic accumulation [4,5]. When this finely tuned balance is disrupted, excess free copper, a redox-active metal, catalyzes the generation of reactive oxygen species (ROS), leading to oxidative stress, mitochondrial dysfunction, DNA damage, and activation of inflammatory signaling pathways, ultimately resulting in cellular injury and death [5,6,7].

In addition to well-established mechanisms such as apoptosis, recent studies have identified a novel copper-dependent form of regulated cell death termed cuproptosis [6]. In this process, copper binds to lipoylated components of the tricarboxylic acid cycle, triggering toxic protein aggregation, loss of iron–sulfur cluster proteins, and severe proteotoxic stress within mitochondria [6]. This mechanism is distinct from apoptosis, necroptosis, and ferroptosis and provides new insights into the molecular basis of copper toxicity.

Early-life exposure to environmental toxicants is of particular concern because developing organisms possess immature detoxification pathways, antioxidant defenses, and organ systems, rendering them more vulnerable to toxic insults [8,9]. According to the Developmental Origins of Health and Disease (DOHaD) hypothesis, adverse exposures during critical developmental windows can induce persistent structural and functional alterations that increase susceptibility to disease later in life [8,9]. Therefore, prenatal and early postnatal exposure to copper sulfate may exert long-lasting effects on multiple organs of juvenile rats through oxidative stress, inflammation, mitochondrial dysfunction, and dysregulated cell death pathways.

Accumulating evidence indicates that excessive copper exposure induces tissue injury in multiple organs, including the spleen, kidneys, and reproductive organs. In the spleen, copper overload has been associated with oxidative stress, apoptosis, DNA damage, and inflammatory responses [10]. In the kidneys, copper sulfate exposure has been shown to trigger oxidative stress, endoplasmic reticulum stress, and apoptosis, resulting in nephrotoxicity [11]. Furthermore, copper can cross the blood–testis barrier and impair male reproductive function through oxidative damage and disruption of spermatogenesis [12].

Despite growing concerns regarding the widespread use of copper-containing compounds, the consequences of early-life copper sulfate exposure on the developing organism remain insufficiently characterized. Therefore, the present study aimed to investigate the toxic effects of copper sulfate pentahydrate exposure on major organs, including the liver, lungs, kidneys, heart, ovaries, and testes, in juvenile male and female rats. Histopathological alterations were assessed using hematoxylin and eosin (H&E) staining. In addition, the immunoreactivity and gene expression levels of the inflammatory markers such as nuclear factor kappa B (NF-κB) and tumor necrosis factor alpha (TNF-α) were evaluated by immunohistochemistry and quantitative real-time PCR (qRT-PCR). Oxidative stress was further assessed by measuring malondialdehyde (MDA), total antioxidant status (TAS), and total oxidant status (TOS) using ELISA-based assays.

## 2. Results

### 2.1. Histological Findings

**Figure 1:**
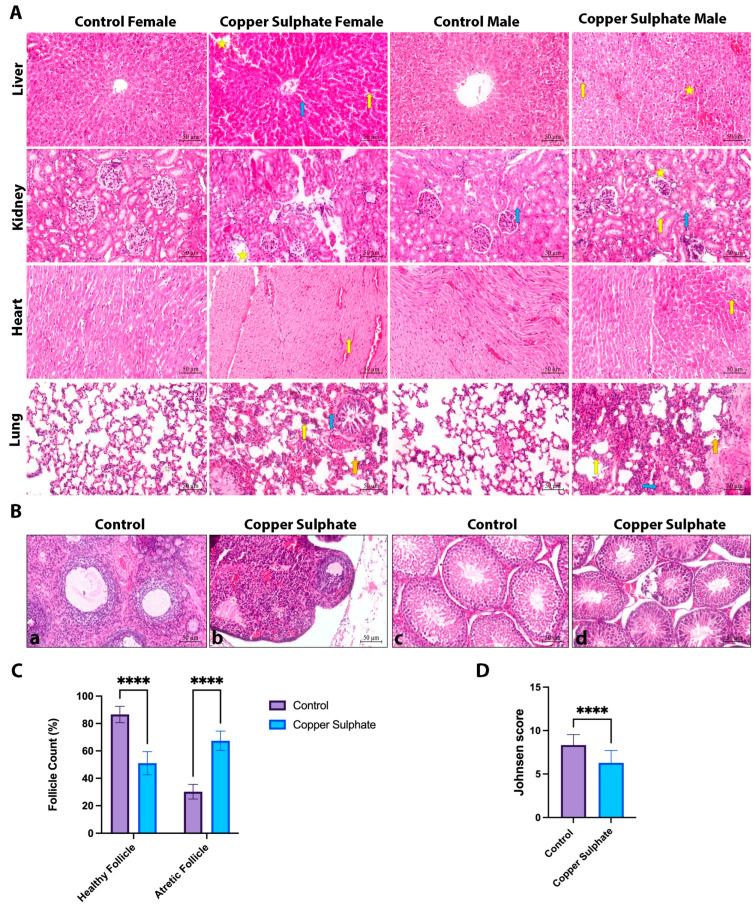
(**A**). Hematoxylin–eosin photomicrographs of liver, kidney, heart and lung tissues of experimental groups. In liver images, yellow arrow indicates pyknotic hepatocytes, blue arrow indicates cell infiltration and star indicates hemorrhage. In kidney images, star indicates glomerular degeneration, yellow arrow indicates vacuolization and blue arrow indicates cell infiltration. In heart images, yellow arrow indicates cell infiltration and blue arrow indicates disarray of myocardial fibers. In lung images, yellow arrow indicates alveolar wall thickening, blue arrow indicates hemorrhage and orange arrow indicates cell infiltration. Magnification 20× and scale bar 50 µm. (**B**). Hematoxylin and eosin staining images of ovarian and testicular tissues. Control ovary (a), copper sulphate ovary (b), control testis (c), copper sulphate testis (d). Magnification is 20× and scale bar is 50 μm. (**C**). Bar graph showing the percentage of healthy and atretic follicles counted in ovarian tissues. Data shown in the graph are expressed as mean ± SD. Statistically, data obtained from the groups were compared using two-way analysis of variance and Sidak’s multiple comparison tests. **** *p* < 0.0001 indicates a significant difference between the control and copper sulfate groups. (**D**). Bar graph showing the Johnsen score determined in testicular tissues. Statistically, data obtained from the groups were compared using independent sample *t*-test. **** *p* < 0.0001 indicates a significant difference between the control and copper sulfate groups.

When the liver sections were examined, it was observed that there were more hepatocytes with pyknotic nuclei and a considerably increased hemorrhage in the copper sulphate groups compared to the control groups. Sinusoidal enlargement was observed especially in copper sulphate female livers and inflammatory cells increased in these spaces (Figure 1A and Figure S1, Table 1).

When the kidney sections were examined, it was observed that there were inflammatory cells in the tubulointerstitial areas and the number of degenerated glomeruli increased in the copper sulphate groups compared to the control groups (Figure 1A and Figure S2, Table 2). Compared to the control male group, increased vacuolization in the tubules was observed in the copper sulphate male group. In copper sulphate female kidney sections, an increase in hemorrhagic areas was observed compared to the control female group (Figure 1A and Figure S2, Table 2).

When the copper-sulphate-treated groups were analyzed, infiltration of inflammatory cells in the heart tissue was observed more in males than females (** *p* = 0.0032). When the control groups were compared within themselves, there were more myocardial fiber irregularities in females than in males (* *p* = 0.0441). In males, inflammatory cell infiltration and irregularity of myocardial fibers were more in the copper sulphate groups compared to the control group (Figure 1A, Table 3).

When both male and female groups were examined, hemorrhage, cellular infiltration and thickening of the alveolar wall were observed more in the copper sulphate group lung sections compared to the control group (Figure 1A and Figure S3, Table 4).

As seen in Figure 1B,C, more atretic follicles were counted in copper sulphate groups compared to the control group. The number of healthy follicles was found to be less than the control group. According to Johnsen scoring system, the seminiferous tubules in the control groups exhibited normal histological architecture, with intact germinal epithelium containing spermatogenic lineage cells and Sertoli cells. In the copper sulphate groups, disruption of the normal seminiferous tubular structure was observed. In addition, loss of spermatogonia was detected in some seminiferous tubules.

### 2.2. Immunohistochemical Findings

**Figure 2:**
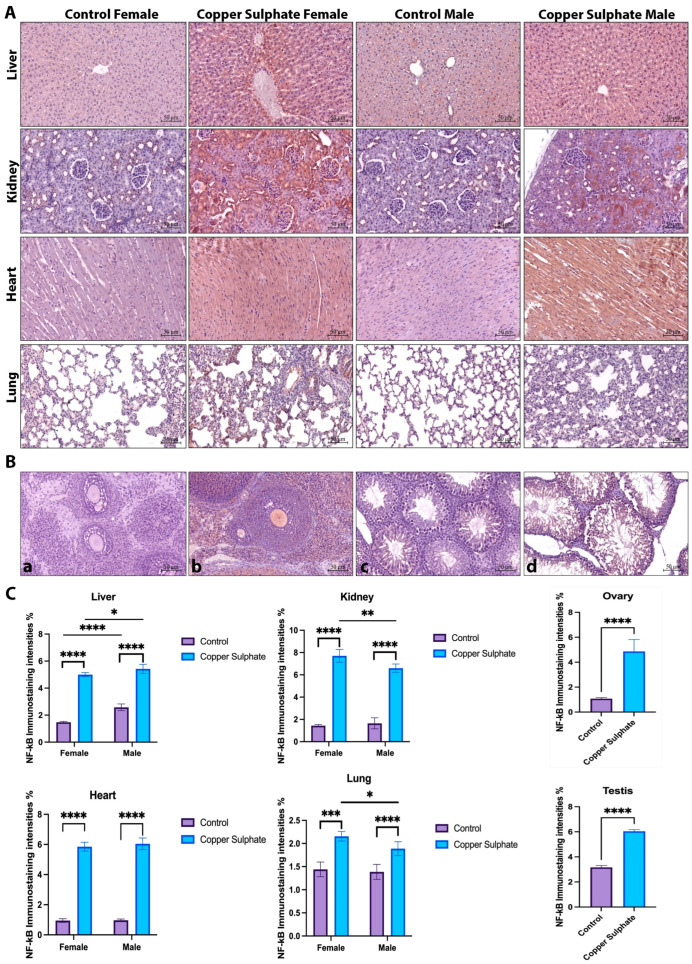
(**A**). NF-kB staining images in liver, kidney, heart and lung tissues of experimental groups. (**B**). NF-kB staining images of ovarian and testicular tissues. Control ovary (a), copper sulphate ovary (b), control testis (c), copper sulphate testis (d). Magnification is 20× and scale bar is 50 μm. (**C**). Bar graphs showing the intensity level of NF-kB immunostaining. Data shown in the graphs of liver, kidney, heart and lung tissues are expressed as mean ± SD. Statistically, the data obtained from the groups were compared using two-way analysis of variance and Sidak’s multiple comparison tests. * *p* < 0.05 and ** *p* < 0.005 indicate a significant difference between females and males in the copper sulfate groups. **** *p* < 0.0001 and *** *p* = 0.0001 indicate a significant difference between the control and copper sulfate groups. Data shown in the graphs of ovarian and testicular tissues are expressed as mean ± SD. Statistically, the data obtained from the groups were compared using independent samples *t* test. **** *p* < 0.0001 indicates a significant difference between the control and copper sulfate groups. Magnification is 20× and scale bar is 50 μm.

NF-kB expression was significantly increased in the tissues of copper sulphate groups compared to control groups (*p* < 0.05, Figure 2). When the copper sulphate groups were compared in terms of sex, it was observed that NF-kB expression was higher in males than females in liver tissue, while it was higher in females in kidney and lung tissues. There was no significant difference between sexes in terms of NF-kB expression in heart tissue (Figure 2). In ovarian and testicular tissues, NF-kB expression was higher in copper sulphate groups (*p* < 0.05, Figure 2).

**Figure 3:**
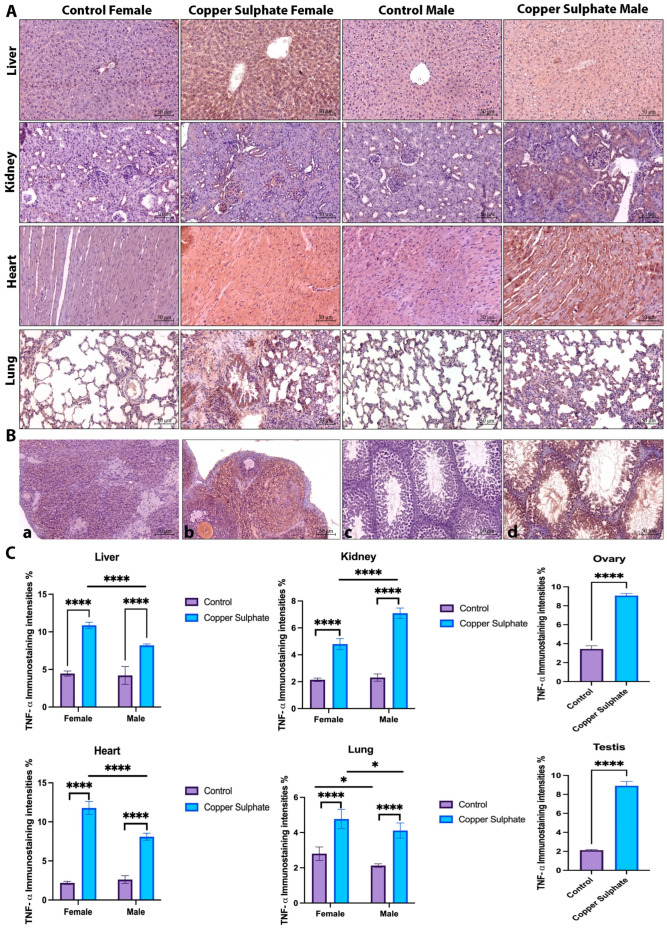
(**A**). TNF-α staining images in liver, kidney, heart and lung tissues of experimental groups. Magnification is 20× and scale bar is 50 μm. (**B**). TNF-α staining images of ovary and testis tissues. Control ovary (a), copper sulphate ovary (b), control testis (c), copper sulphate testis (d). Magnification is 20× and scale bar is 50 μm. (**C**). Bar graphs showing the intensity level of TNF-α immunostaining. Data shown in the graphs of liver, kidney, heart and lung tissues are expressed as mean ± SD. Statistically, the data obtained from the groups were compared using two-way analysis of variance and Sidak’s multiple comparison tests. **** *p* < 0.0001 and * *p* < 0.05 indicate a statistically significant difference between males and females in copper sulfate groups. **** *p* < 0.0001 indicates a significant difference between the control and copper sulfate groups. Data shown in the graphs of ovarian and testicular tissues are expressed as mean ± SD. Statistically, the data obtained from the groups were compared using independent samples *t*-test. **** *p* < 0.0001 indicates a significant difference between the control and copper sulfate groups. Magnification is 20× and the scale bar is 50 μm.

It was observed that TNF-α expression increased in the tissues examined in copper sulphate groups compared to the control group (*p* < 0.05, Figure 3). When copper sulphate groups were compared in terms of sex, it was observed that TNF-α expression was higher in females in heart, lung and liver tissues, while it was higher in males in kidney tissues. Increased expression intensity in ovary and testis tissues was also observed in copper sulphate groups (*p* < 0.05, Figure 3).

### 2.3. ELISA Findings

**Figure 4:**
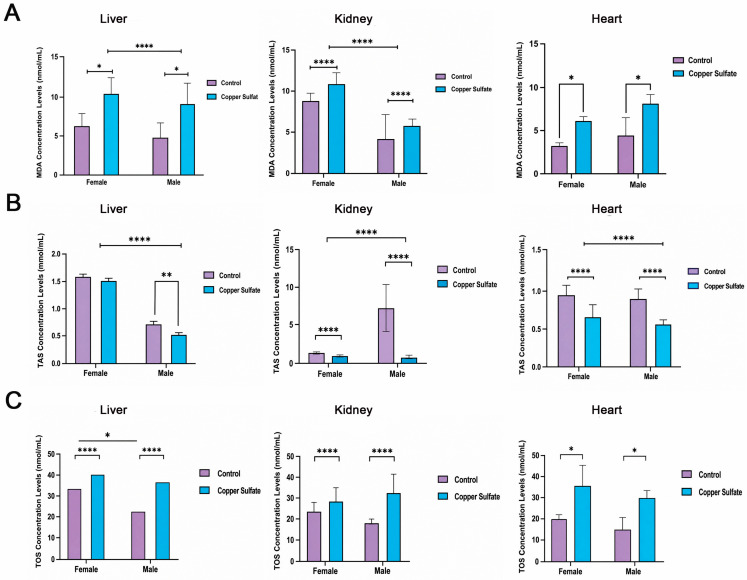
Concentration levels of malondialdehyde (MDA) (**A**), total antioxidant status (TAS) (**B**), and total oxidant status (TOS) (**C**) in liver, kidney, and heart tissues of the experimental groups. Data are presented as mean ± SD. Statistical analyses were performed using two-way ANOVA followed by Tukey’s post hoc test. * *p* < 0.05, ** *p* < 0.01, and **** *p* < 0.0001 were considered statistically significant. MDA: malondialdehyde; TAS: total antioxidant status; TOS: total oxidant status.

**Figure 5:**
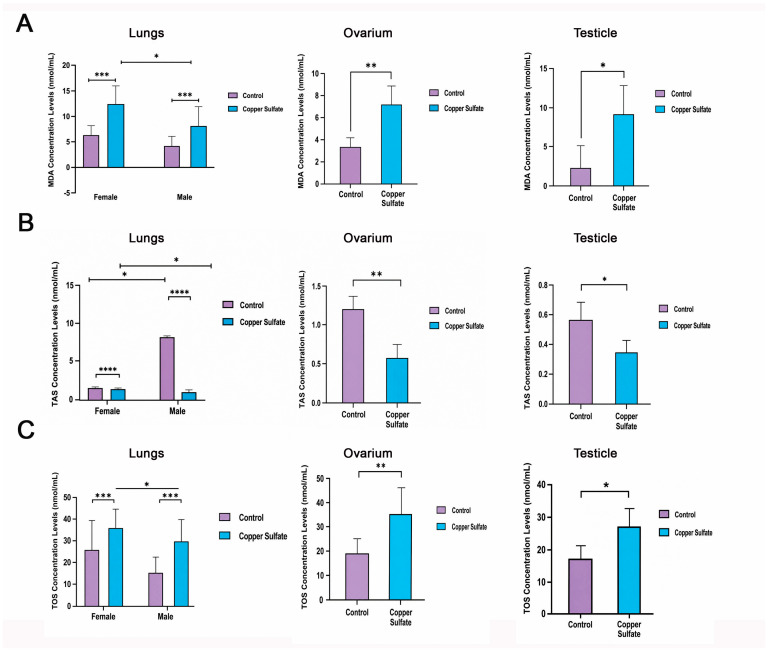
Concentration levels of malondialdehyde (MDA) (**A**), total antioxidant status (TAS) (**B**), and total oxidant status (TOS) (**C**) in lung, ovary, and testicle tissues of the experimental groups. Data are presented as mean ± SD. Statistical analyses for lung tissues were performed using two-way ANOVA followed by Tukey’s post hoc test, whereas comparisons in ovary and testicle tissues were analyzed using the independent samples *t*-test. * *p* < 0.05, ** *p* < 0.01, *** *p* < 0.001, and **** *p* < 0.0001 were considered statistically significant. MDA: malondialdehyde; TAS: total antioxidant status; TOS: total oxidant status.

MDA concentration levels were significantly higher in the tissues of copper sulphate groups compared to control groups (*p* < 0.05, Figure 4 and Figure 5). When the control and copper sulphate groups were compared in terms of sex, it was observed that MDA levels were higher in females than males in kidney tissue and higher in males than females in heart tissue (Figure 4 and Figure 5). TAS (Total Antioxidant Level) concentration levels were significantly higher in the tissues of control groups compared to copper sulphate groups (*p* < 0.05, Figure 4 and Figure 5). When the control and copper sulphate groups were compared in terms of sex, it was observed that TAS levels were higher in females than males in liver tissue. In addition, it was observed that TAS levels of the control male group were significantly higher in lung and kidney tissues compared to the other groups (Figure 4 and Figure 5). TOS (Total Oxidant Level) concentration levels were significantly higher in the tissues of copper sulphate groups compared to the control groups (*p* < 0.05, Figure 4 and Figure 5). When the control and copper sulphate groups were compared in terms of sex, it was observed that TOS levels were higher in females than males in liver, heart and lung tissues (Figure 4 and Figure 5).

### 2.4. qRT-PCR Findings

#### mRNA Analysis Nf-κB and TNF-α

**Figure 6:**
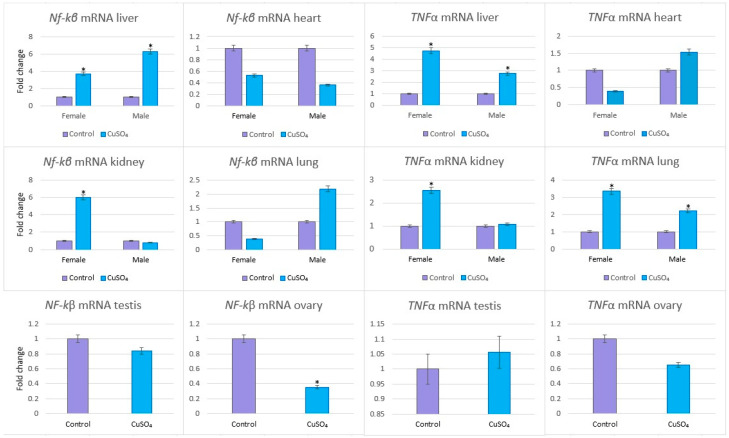
The results of real-time PCR analysis. Relative mRNA expression of Nf-κβ gene and TNFα in liver, heart, kidney, lung ovary and testis tissues in males and females exposed to CuSO_4_ were given as fold change compared to the related controls. * Represents the significance of *p* < 0.001 compared to control. GAPDH was used as the reference gene for normalization.

The mRNA levels of the Nf-κB gene were changed in livers (3.68*- and 6.28-fold change, male and female respectively), in hearts (0.53*- and 0.36*-fold change), in kidneys (6.02*- and 0.78*-fold change) and in lungs (0.38- and 2.19-fold change) compared to mRNA levels of related control tissues (* *p* < 0.001) (Figure 6). The mRNA levels of the TNFα gene were changed in livers (4.72*- and 2.75*-fold change; male and female respectively), in hearts (0.39- and 1.54-fold change), in kidneys (2.55*- and 1.07-fold change) and in lungs (3.35*- and 2.22*-fold change) compared to mRNA levels of related control tissues (* *p* < 0.001) (Figure 6). The mRNA levels of the Nf-κB and TNFα gene were changed testis tissues compared to the control testis (0.84- and 1.06-fold change, respectively). The mRNA levels of the Nf-κB and TNFα gene were changed ovary tissues compared to the control ovaries (0.36*- and 0.65-fold change, respectively) (* *p* < 0.001) (Figure 6).

## 3. Discussion

Copper, the third most abundant trace element in humans, is essential for various biological processes, including those in the respiratory, immune, reproductive, endocrine, and central nervous systems [1,3,13].

The excess of free Cu causes intracellular and extracellular Cu accumulation in various organs and has a negative effect due to its ability to produce free radical species leading to oxidative stress [4]. Although chronic Cu exposure has become an increasingly important public health problem due to its increasing use, its early effects are largely unknown. Based on these data, we aimed to determine the possible damage to vital organs such as liver, lung, kidney, heart, ovary and testis in male and female rat pups exposed to copper sulphate. In an experimental study [14], it was shown that antioxidant (GSH, TAC) and oxidative stress (MDA) levels in liver, kidney and brain tissue exposed to different doses of CuSO_4_ for different time periods increased and this exposure caused dysfunction in the relevant organs [14]. Low GSH level, total antioxidant capacity (TAC) and high MDA level and other oxidative stress markers have been shown in previous studies, for example, in brain and liver tissue in experimental ‘Cu toxicity’ models [4,15,16,17,18]. In our study, unlike similar studies, we investigated tissue damage and oxidative stress markers in pups instead of adult rats and in comparison between sexes, and we showed that copper sulphate exposure increased MDA and TOS levels and decreased TAS levels in all tissues, similar to the literature. Again, in accordance with the literature, we observed that histopathological damage was observed in all tissues and inflammatory cytokine levels were similarly increased compared to control groups.

In a study, epithelial hyperplasia, alveolar histiocytosis and neutrophil increase were observed in the lungs of rats exposed to copper sulphate by inhalation. In our study, we observed hemorrhage, cellular infiltration and thickening of the alveolar wall in the copper sulphate group lung sections in both males and females compared to the control group [13]. In another study, they investigated Cu concentrations in the liver, brain and kidney in a rat model after chronic exposure to oral copper sulphate at different subtoxic doses and reported that Cu concentration was highest in the liver (29-fold), followed by kidney (3-fold) and brain (1.5-fold). They reported that chronic oral exposure to subtoxic levels of copper sulphate in rats caused neurobehavioural abnormalities and liver and kidney dysfunctions due to increased Cu concentration in the relevant organs and that the liver was the most vulnerable organ in this process [14].

Cisternas et al. [19] reported that subchronic Cu exposure altered growth curves in young rats. Consistent with these findings, our results demonstrated that copper sulfate caused early hepatic morphological changes copper sulphate caused early hepatic morphological changes and an increase in Kupffer cell-dependent respiratory burst in parallel with the activation of NF-kB activity as proved by light and electron microscopy, in our study, a higher number of hepatocytes with pyknotic nuclei and a considerably increased hemorrhage were observed in the liver tissue in the copper sulphate groups compared to the control groups. Sinusoidal enlargement was observed especially in the livers of copper sulphate females and inflammatory cells increased in these spaces. In addition, NF-kB immunoreactivity was quite high in copper sulphate groups [19]. Although previous studies on Cu have generally focused on its effects on the liver, kidneys and spleen [20,21], recent studies have focused on its effects on reproductive functions and reproductive system.

Reproductive toxicity is one of the effects of Cu as reported in various in vivo and in vitro experiments [22,23,24,25]. The effects of Cu on male fertility include a decrease in spermatozoa number, motility and viability [26]. Studies have shown that Cu intake, even at low doses, causes adverse effects on testicular morphology in male mice [27].

Although the molecular mechanisms of the effects of Cu on male fertility are not fully understood, it can be suggested that this is due to excessive reactive oxygen species (ROS) and oxidative stress [28]. In their study, Sarawi et al. [28] showed that oral supplementation of CuSO_4_ caused degeneration of seminiferous tubules and loss of spermatogenic series and complete absence of mature spermatozoa. In our study, when evaluated according to the Johnsen scoring system, it was observed that the interstitial area containing Leydig cells and the germinal epithelium containing spermatogenic line cells and Sertoli cells had normal morphology in the control groups, while the normal structure of the seminiferous tubules was disrupted in the copper sulphate groups and spermatogonium loss was observed in some seminiferous tubules. It was also shown that NF-kB expression increased. Copper (Cu) negatively impacts male reproductive functions, as evidenced by reductions in sperm concentration, viability, and motility. Copper nanoparticles (CuNPs) have been shown to induce oxidative stress in vitro, which contributes to reproductive toxicity. The toxic effects of CuNPs are more pronounced in male mice compared to females. Although further studies are necessary to draw firm conclusions, it is evident that Cu disrupts reproductive systems in both genders and inhibits embryo development in a dose-dependent manner [29]. For instance, a study reported a reduction in antral follicle numbers in mouse ovaries after 14 days of exposure to 100 mg/kg copper sulfate (CuSO_4_). Prolonged exposure (35 days at 200 mg/kg) led to significant decreases in all follicular types, including primordial, primary, growing, secondary, and antral follicles, as well as the corpus luteum [27]. Another study found that copper exposure reduced the number of preantral and antral follicles while increasing the number of atretic follicles. They also suggested that copper-induced apoptosis in ovarian granulosa cells, mediated by the caspase-dependent pathway, and miRNAs may play a role in these effects [30]. Similarly, we observed more atretic follicles in the copper sulphate groups than in the control group. We found that the number of healthy follicles was less than in the control group and again showed that oxidative stress increased NF-kB immunoreactivity in ovarian tissue as in all tissues.

## 4. Materials and Methods

### 4.1. Procedures Performed on Animals

Permission was obtained from Erciyes University Animal Experiments Local Ethics Committee (HADYEK) for this study (Protocol Number: 23/063, Approval Date: 16 May 2023). Experimental procedures were performed in accordance with the guidelines for the care and use of laboratory animals in scientific research. Male and female Sprague–Dawley rats, 30–40 days old, weighing 50–70 g, were used in the study. The rats were fed with standard rat diet. Copper sulphate [31] at a dose of 100 mg/kg was administered via oral gavage to the copper sulphate group, while the control group received saline via oral gavage. At the end of the 14th day, animals were anesthetized via intramuscular injection with xylazine at a dose of 15 mg/kg and ketamine at a dose of 60 mg/kg. Animals were euthanized by cervical dislocation in accordance with institutional ethical guidelines, tissues were taken and histological, immunohistochemical, molecular and biochemical examinations were performed.

Control Group (*n* = 5 male, *n* = 5 female); animals in this group received 1 mL of saline solution via oral gavage.

Copper Sulphate Group (*n* = 5 male, *n* = 5 female); animals in this group were given copper sulphate dissolved in saline at a dose of 100 mg/kg/day by oral gavage [31,32].

### 4.2. Histological Analyses

Liver, kidney, heart, lung, testicles, and ovary tissues were fixed with 10% formaldehyde, washed overnight in tap water, and dehydrated through a graded alcohol series (70%, 80%, 90%, and 100%). The samples were clarified in xylene (Merck, Darmstadt, Germany; Cat. No. 1082984000) and embedded in paraffin. The hematoxylin and eosin (H&E) staining procedure was performed as follows. Tissue sections mounted on slides were incubated overnight in a 58 °C oven to ensure adequate adhesion and spreading of the tissue on the slide surface. Subsequently, the sections were deparaffinized by immersion in xylene I and xylene II for 10 min each. The sections were then rehydrated through a descending ethanol series (100%, 96%, 80%, and 70%) for 5 min each and washed under running tap water for 2 min. Thereafter, the sections were stained with Harris hematoxylin for 8 min and rinsed under running water for 2 min. To obtain a pale blue coloration, the sections were briefly differentiated in acid alcohol for 1 s and subsequently washed under running water for 1 min. Following washing, the sections were counterstained with eosin for 10 min. Excess eosin was removed by passing the sections through an ascending ethanol series (70%, 80%, 96%, and 100%) for 5 min each. Finally, the sections were cleared in xylene I and xylene II for 5 min each and mounted using Entellan mounting medium (Merck, Darmstadt, Germany; Cat. No. 107961.0100). Histomorphological evaluations were conducted under a Zeiss Axiscope 3 light microscope (Carl Zeiss, Oberkochen, Germany) and documented using a Colibri 3 digital camera (Carl Zeiss, Oberkochen, Germany). In liver samples, hollow and pyknotic hepatocytes, sinusoidal dilation, hemorrhage, and inflammatory cell infiltration in venules and sinusoids were analyzed [33]. Kidney sections were assessed for glomerular degeneration and tubulointerstitial damage, including vacuolization, hemorrhage, and inflammatory cell infiltration [34]. Heart tissues were evaluated for inflammatory cell infiltration and myocardial fiber disorganization [35,36]. Lung tissue analysis focused on hemorrhage, cellular infiltration, and alveolar wall thickening [37]. Histopathological scoring of all tissues was performed on a scale of 0 to 3, with two independent, blinded observers evaluating at least 10 images per rat per experimental group (10 rats: 5 male, 5 female). Testicular histology was scored using the Johnsen scoring system [38,39]. Ovarian sections were examined every fifth slice to quantify healthy and atretic follicles. Follicles with irregular plasma membranes, eosinophilic oocytes, or granulosa cells with pyknotic nuclei were categorized as atretic, while healthy follicles were classified according to Pederson’s criteria [36,40].

### 4.3. Immunohistochemical Analyses

Immunohistochemical analysis of NF-kB (BT-MCA1291; Bioassay Technology Laboratory, Shanghai, China) and TNF-α (E-AB-22159; Elabscience, Wuhan, China) was conducted on 5-μm sections of liver, kidney, heart, lung, testis, and ovary tissues from experimental groups using the Avidin-Biotin peroxidase method [41,42]. After deparaffinization, citrate buffer (pH 6.0; Thermo Fisher Scientific, Loughborough, UK, AP-9003-500) was used for epitope retrieval. Endogenous peroxidase activity was blocked with 3% hydrogen peroxide in methanol, and Ultra V block solution (Thermo Fisher Scientific, Loughborough, UK, TA-125-UB) minimized non-specific staining. Sections were incubated overnight at 4 °C with primary antibodies (NF-kB, 1:50; TNF-α, 1:100), followed by 40 min treatment with biotinylated goat anti-polyvalent secondary antibody (Thermo Fisher Scientific, Loughborough, UK, TP-125-BN) at room temperature. After PBS washes, streptavidin peroxidase (Thermo Fisher Scientific, Loughborough, UK, TS-125-HR) was applied for 30 min, and the antibody complex was visualized with diaminobenzidine (DAB) chromogen (Thermo Fisher Scientific, Loughborough, UK, TA-125-HD). Counterstaining was performed with Gill III hematoxylin (Merck, Darmstadt, Germany; Cat. No. 1.05174.1000). Dehydration was achieved using an alcohol gradient, and slides were mounted with Entellan. Tissue sections were analyzed under a Zeiss Axiscope 3 light microscope and imaged using a Colibri 3 digital camera. Immunoreactivity levels were assessed using Fiji/ImageJ software (version 1.46; NIH, Bethesda, MD, USA).

### 4.4. ELISA Analyses

MDA (Cat. No. 201-11-0157; Sunred Biological Technology, Shanghai, China), TAS (Rel Assay Diagnostics, Gaziantep, Türkiye) and TOS (Rel Assay Diagnostics, Gaziantep, Türkiye) analyses of oxidative stress markers in tissues were also evaluated by ELISA method and analyses were performed according to the purchased commercial kit procedures. Samples for MDA were measured at 450 nm, for TAS at 660 nm and for TOS at 530 nm wavelength using a SPECTROstar Nano microplate reader (BMG Labtech, Ortenberg, Germany) according to the kit protocol and the data were expressed in nmol/mL.

### 4.5. Genetic Analyses

Total RNA was isolated from the heart, lung, liver, kidney, ovary, and testis tissues of rats using PureZole reagent (Bio-Rad, Hercules, CA, USA) following the manufacturer’s instructions. The concentration and purity of RNA were measured using a Nanodrop ND-1000 spectrophotometer (v3.7; Thermo Fisher Scientific, Wilmington, DE, USA). The RNA samples were stored at −80 °C until further use. For cDNA synthesis, 1 μg of total RNA from each sample was reverse-transcribed using the iScript Reverse Transcription Supermix (Bio-Rad, Hercules, CA, USA). mRNA expression levels of NF-kβ and TNF-α were analyzed using Real-Time PCR on a Step-One-Plus Thermocycler (Applied Biosystems, Foster City, CA, USA). The reaction was performed in a 20 μL mixture containing cDNA, site-specific primers (Oligomer Biotechnology, Ankara, Türkiye), SsoAdvanced Universal Inhibitor-Tolerant SYBR Green Supermix (Bio-Rad, Hercules, CA, USA), and nuclease-free water. GAPDH served as the internal reference gene. Primer sequences were designed as mentioned in [43] for TNF-α and were designed as mentioned in [44] for Nf-κB: Rat-Nf-κB F: 5′- GCAAACCTGGGAATACTTCATGTGACTAAG-3′, Rat-Nf-κB R: 5′-ATAGGCAAGGTCAGAATGCACCAGAAGTCC-3′, Rat-TNF-α F: 5′ AAATGGGCTCCCTCTCATCAGTTC-3′, Rat-TNF-α R: 5′- TCTGCTTGGTGGTTTGCTACGAC-3′, Rat-GAPDH F: 5′- GAGGACCAGGTTGTCTCCTG-3′, Rat-GAPDH R: 5′-GGATGGAATTGTGAGGGAGA- 3′.

### 4.6. Statistical Analyses

The analyses of the findings obtained as a result of immunohistochemical staining were measured using Image J Version 1.46 software. All data were expressed as mean ± SD and analyzed by One-way ANOVA test and Tukey’s post hoc test for parametric tests. For non-parametric tests, median minimum–maximum values and Independent Sample *t*-test were applied and *p* < 0.05 was accepted as significant in the analyses. All the data analyses for qRT-PCR were performed using REST 2009 V2.0.13 [45] where *p* < 0,05 is deemed to represent a statistically significant result.

## 5. Conclusions

In conclusion, early-life exposure to copper sulphate pentahydrate was found to exert significant toxic effects on multiple organ systems in juvenile rats, particularly the liver, kidney, heart, lung, and reproductive tissues. Biochemical analyses revealed marked alterations in MDA, TAS, and TOS levels, indicating pronounced oxidative stress, while elevated NF-κB and TNF-α concentrations confirmed concurrent activation of pro-inflammatory pathways. Together, these findings suggest that oxidative damage and inflammation are central mechanisms underlying the histopathological lesions observed across the affected organs. The data further indicate that excessive copper exposure during critical developmental windows may predispose individuals to progressive organ dysfunction and long-term systemic toxicity. Given the widespread agricultural application of copper-based compounds, these results carry meaningful implications for public health policy, particularly regarding permissible exposure limits in early childhood. Future studies should aim to elucidate the molecular mechanisms driving copper-induced toxicity more precisely, with particular attention to recently characterized pathways such as cuproptosis. Such mechanistic insight will not only deepen our understanding of copper-related pathology but also provide a scientific basis for developing targeted therapeutic strategies and evidence-based regulatory frameworks within the broader context of environmental health and chemical safety.

## Figures and Tables

**Table 1:** Histopathological damage rates in liver tissues of the experimental groups.

Groups	Pyknotic Hepatocyte	Sinusoidal Dilatation	Hemorrhage	Inflammatory Cell Infiltration
Control Female		0.4 ± 0.55	0.2 ± 0.45	0.4 ± 0.55
Copper Sulphate Female	2.6 ± 0.55 ****	2.4 ± 0.89 ***	2 ± 0.71 ***	2 ± 0.71 **
Control Male	0.8 ± 0.45	0.8 ± 0.84	0.6 ± 0.55	1 ± 0.71
Copper Sulphate Male	2.8 ± 0.45 ****	1.6 ± 0.55	2.6 ± 0.55 ****	1.8 ± 0.45

Statistically, the data obtained from the groups were compared using two-way analysis of variance and Sidak’s multiple comparison tests. The data shown in the table are expressed as mean ± SD. **** *p* < 0.0001, *** *p* = 0.0003, and ** *p* = 0.0016 indicate a statistically significant difference compared to the control group.

**Table 2:** Histopathological damage rates in kidney tissues of the experimental groups.

Groups	Vacuolization	Hemorrhage	Inflammatory Cell Infiltration	Glomerular Degeneration
Control Female	1 ± 0.71	0.2 ± 0.45	0.4 ± 0.55	0.8 ± 0.83
Copper Sulphate Female	2 ± 0.71	1.2 ± 0.45 *	2.2 ± 0.45 ***	2 ± 0.71 *
Control Male	0.8 ± 0.45	1 ± 0.71	0.6 ± 0.55	0.6 ± 0.55
Copper Sulphate Male	2.6 ± 0.89 **	1.2 ± 0.45	1.6 ± 0.89 *	2.6 ± 0.55 ***

Statistically, the data obtained from the groups were compared using two-way analysis of variance and Sidak’s multiple comparison tests. The data shown in the table are expressed as mean ± SD. *** *p* < 0.005, ** *p* = 0.002, and * *p* < 0.05 indicate a statistically significant difference compared to the control group.

**Table 3:** Histopathological damage rates in heart tissues of the experimental groups.

Groups	Inflammatory Cell Infiltration	Dysregulation of Myocardial Fibers
Control Female	0.6 ± 0.54	1.2 ± 0.44
Copper Sulphate Female	1.2 ± 0.45	1.6 ± 0.55
Control Male	0.8 ± 0.44	0.4 ± 0.54
Copper Sulphate Male	2.4 ± 0.54 ***	2.2 ± 0.45 ****

Statistically, the data obtained from the groups were compared using two-way analysis of variance and Sidak’s multiple comparison tests. The data shown in the table are expressed as mean ± SD. *** *p* = 0.0002, **** *p* < 0.0001 indicates a statistically significant difference compared to the control group.

**Table 4:** Histopathological damage rates in lung tissues of the experimental groups.

Groups	Hemorrhage	Cellular Infiltration	Thickening of the Alveolar Wall
Control Female	1.2 ± 0.44	0.8 ± 0.45	0.2 ± 0.45
Copper Sulphate Female	2.6 ± 0.54 **	2.4 ± 0.89 **	2.2 ± 0.84 **
Control Male	1 ± 0.70	0.4 ± 0.55	1.2 ± 0.84
Copper Sulphate Male	2.4 ± 0.55 **	2.4 ± 0.89 ***	2.6 ± 0.90 *

Statistically, the data obtained from the groups were compared using two-way analysis of variance and Sidak’s multiple comparison tests. The data shown in the table are expressed as mean ± SD. *** *p* = 0.0001, ** *p* < 0.005, * *p* < 0.05 indicates a statistically significant difference compared to the control group.

## Data Availability

The data presented in this study are available on request from the corresponding authors.

## Supplementary Materials

The following supporting information can be downloaded at: https://www.mdpi.com/article/10.3390/ijms27125542/s1.

## Abbreviations

The following abbreviations are used in this manuscript:

ANOVA	Analysis of Variance
cDNA	Complementary DNA
Cu	Copper
CuSO_4_	Copper sulfate
DAB	Diaminobenzidine
ELISA	Enzyme-Linked Immunosorbent Assay
GAPDH	Glyceraldehyde-3-phosphate dehydrogenase
H&E	Hematoxylin and Eosin
HADYEK	Animal Experiments Local Ethics Committee
IHC	Immunohistochemistry
MDA	Malondialdehyde
mRNA	Messenger RNA
NF-κB	Nuclear factor kappa B
PBS	Phosphate-buffered saline
PCR	Polymerase Chain Reaction
qRT-PCR	Quantitative Real-Time Polymerase Chain Reaction
ROS	Reactive Oxygen Species
SD	Standard Deviation
TAS	Total Antioxidant Status
TNF-α	Tumor Necrosis Factor-alpha
TOS	Total Oxidant Status
