# Supplementary material for: Evaluation of the Effect of Copper Sulfate Exposure on Organs in Juvenile Rats
Source: Int J Mol Sci. 2026 Jun 19;27(12):5542. doi: 10.3390/ijms27125542 (PMC13300441; doi:10.3390/ijms27125542)
Supplement: Supplementary file 1 [file ijms-27-05542-s001.zip › ijms-4316186-supplementary.pdf]

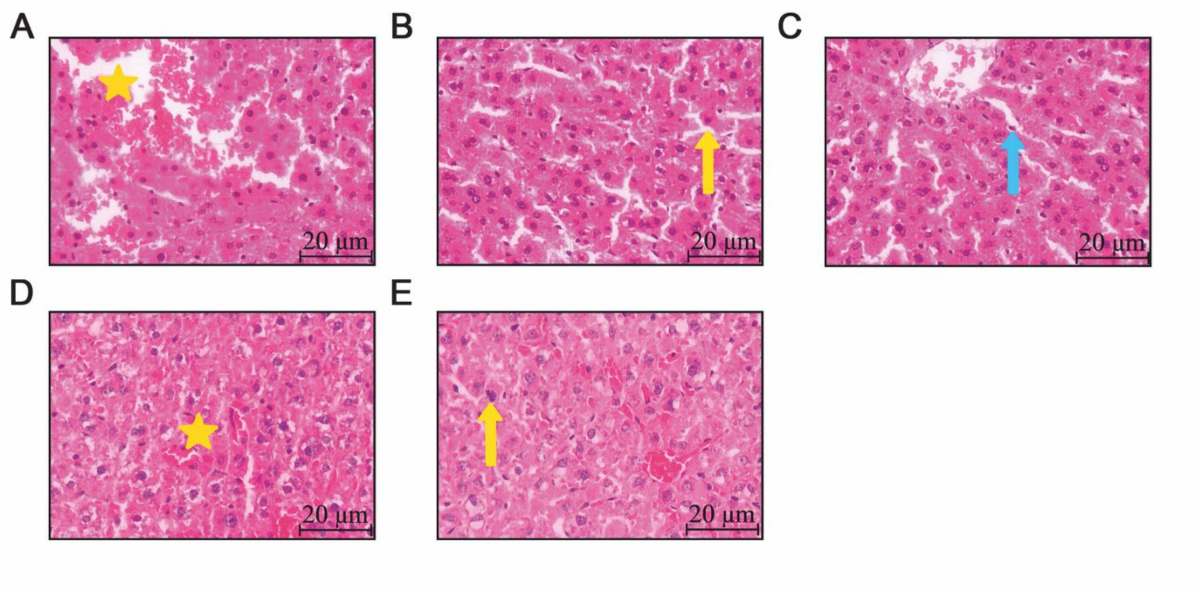

Supplementary Figure S1. Representative hematoxylin and eosin stained photomicrographs of liver tissues from copper sulfate-treated groups. Copper sulfate female liver tissues are shown in panels (A–C), whereas copper sulfate male liver tissues are shown in panels (D–E). Yellow arrows indicate pyknotic hepatocytes, blue arrows indicate inflammatory cell infiltration, and yellow stars indicate hemorrhage. Images were obtained at 40× magnification. Scale bar = 20 µm.

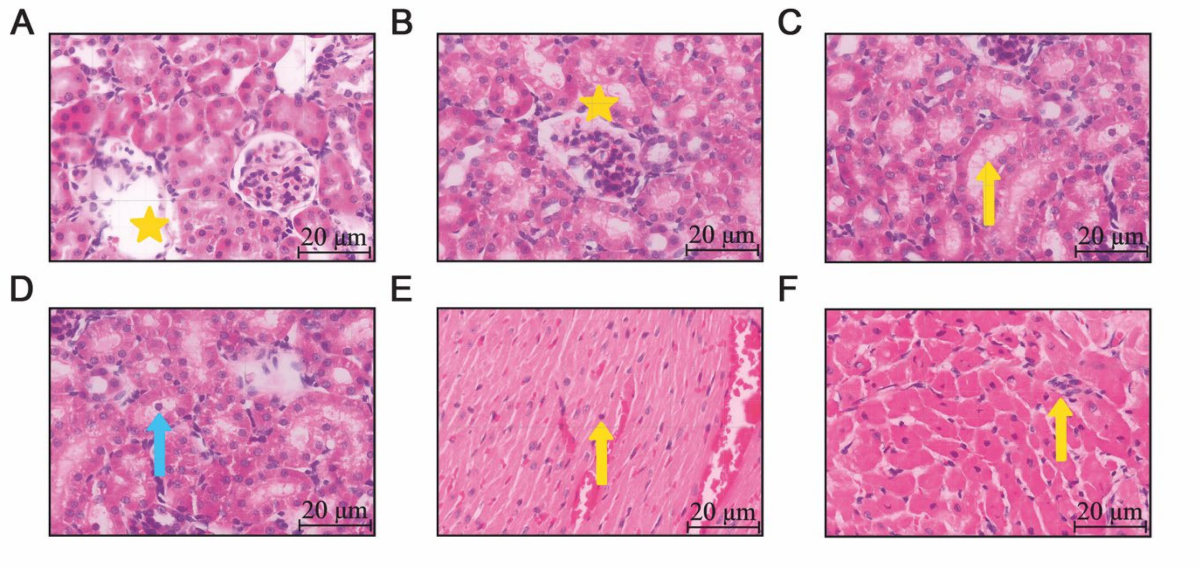

Supplementary Figure S2. Representative hematoxylin and eosin-stained photomicrographs of kidney and heart tissues from copper sulphate-treated groups. Copper sulphate female kidney tissues are shown in panel (A), whereas copper sulphate male kidney tissues are shown in panels (B–D). Copper sulphate female heart tissues are shown in panel (E), whereas copper sulphate male heart tissues are shown in panel (F). In kidney images, yellow stars indicate glomerular degeneration, yellow arrows indicate vacuolization, and blue arrows indicate inflammatory cell infiltration. In heart images, yellow arrows indicate inflammatory cell infiltration. Images were obtained at 40× magnification. Scale bar = 20 µm.

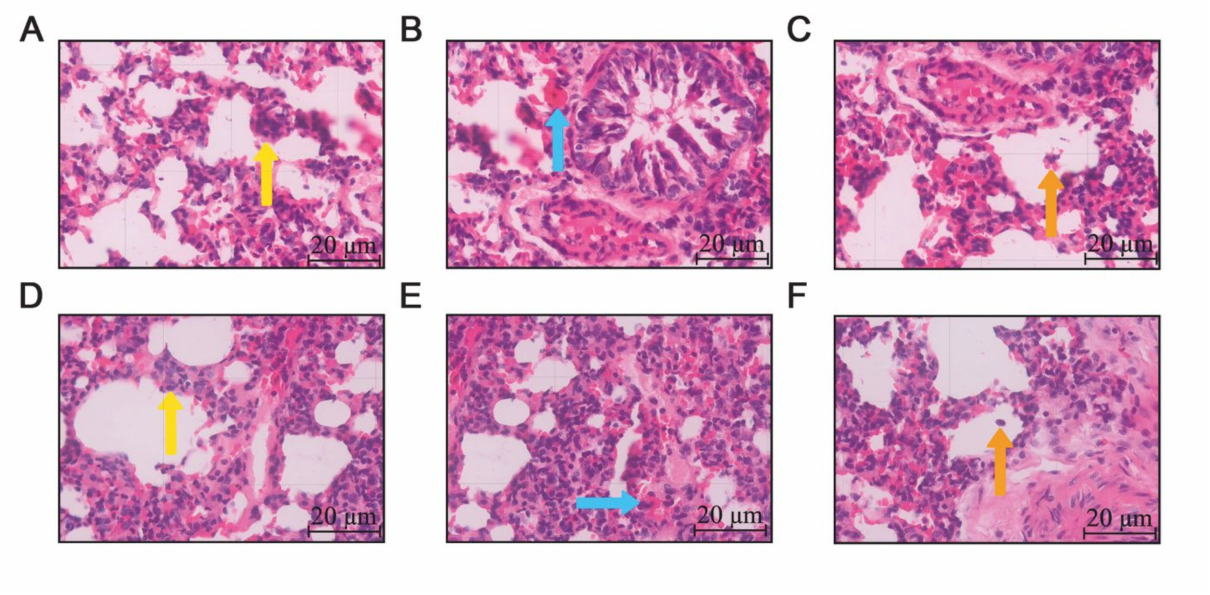

Supplementary Figure S3. Representative hematoxylin and eosin-stained photomicrographs of lung tissues from copper sulphate-treated groups. Copper sulphate female lung tissues are shown in panels (A–C), whereas copper sulphate male lung tissues are shown in panels (D–F). Yellow arrows indicate alveolar wall thickening, blue arrows indicate hemorrhage, and orange arrows indicate inflammatory cell infiltration. Images were obtained at 40× magnification. Scale bar = 20 μm.
